# Pancreatobiliary versus intestinal histologic type of differentiation is an independent prognostic factor in resected periampullary adenocarcinoma

**DOI:** 10.1186/1471-2407-8-170

**Published:** 2008-06-11

**Authors:** Arne Westgaard, Svetlana Tafjord, Inger N Farstad, Milada Cvancarova, Tor J Eide, Oystein Mathisen, Ole Petter F Clausen, Ivar P Gladhaug

**Affiliations:** 1 Faculty of Medicine, University of Oslo, Rikshospitalet University Hospital, 0027 Oslo, Norway; 2 Department of Surgery, Rikshospitalet University Hospital, 0027 Oslo, Norway; 3 Pathology Clinic, Rikshospitalet University Hospital, 0027 Oslo, Norway; 4 Biostatistics, Rikshospitalet University Hospital, 0027 Oslo, Norway

## Abstract

**Background:**

Resectable adenocarcinomas in the pancreatic head, by definition "periampullary", originate from ampullary, duodenal, biliary, or ductal pancreatic epithelium. Typically, periampullary adenocarcinomas have either intestinal or pancreatobiliary type of differentiation, and the type of differentiation might be prognostically more important than the anatomic site of origin. The aim of the study was to determine whether the histologic type of differentiation is an independent prognostic factor in periampullary adenocarcinoma, and whether tumour origin predicts the prognosis in pancreatobiliary type carcinomas independently of resection margin involvement, tumour size, nodal involvement, perineural and vascular infiltration, and degree of differentiation.

**Methods:**

Histopathologic variables in 114 consecutively resected periampullary adenocarcinomas of pancreatobiliary (n = 67) and intestinal (n = 47) type differentiation were evaluated using a standardized, systematic protocol for evaluation of the resected specimen (study group). Histologic type of differentiation and tumour origin were compared as predictors of survival, and the results were validated by comparison with a historical control group consisting of 99 consecutive pancreaticoduodenectomies performed before standardization of histopathologic evaluation. Associations between histopathologic variables were evaluated by Chi-square and Mann-Whitney tests. Survival was estimated by the Kaplan-Meier method, comparing curves using log-rank test, and by univariate and multivariable Cox regression analysis.

**Results:**

Both in the study group (n = 114) and in the historical control group (n = 99), the histologic type of differentiation independently predicted survival, while tumour origin predicted survival only in univariate analysis. Independent adverse predictors of survival in the study group were pancreatobiliary type differentiation (p < 0.001; HR 3.1; CI 1.8–5.1), regional lymph node involvement (p < 0.001; HR 2.5; CI 1.5–4.4), vessel involvement (p = 0.012; HR 1.9; CI 1.2–3.1), and increasing tumour diameter (measured in cm, p = 0.011; HR 1.3; CI 1.1–1.5). For pancreatobiliary differentiated adenocarcinomas (n = 67), lymph node status, vessel involvement, and tumour diameter remained independent prognostic factors, while tumour origin did not independently predict the prognosis due to significant association with tumour size (p < 0.001) and lymph node involvement (p = 0.004).

**Conclusion:**

Pancreatobiliary versus intestinal type of differentiation independently predicts poor prognosis after pancreaticoduodenectomy for periampullary adenocarcinoma. Lymph node involvement, vessel infiltration, and increasing tumour diameter are adverse predictors of survival in tumours with pancreatobiliary differentiation.

## Background

Resectable primary adenocarcinomas located in the pancreatic head may derive from the pancreas, the ampulla, the distal bile duct, or the duodenum. Collectively, these tumours may be referred to as "periampullary" adenocarcinomas, of which those originating from the pancreas have the worst prognosis [1]. The histopathologic and biologic features associated with ductal pancreatic adenocarcinoma are different from non-pancreatic periampullary tumours [2], and it has thus been customary to consider these four subtypes of periampullary adenocarcinoma as separate entities.

The precise origin of a periampullary adenocarcinoma is often difficult to determine even with standardized histopathologic evaluation, particularly if the tumour is large and involves more than one potential site of origin [3-8]. Tumour destruction of normal periampullary anatomy [9], and presence of epithelial dysplasia in more than a single periampullary compartment, occurs frequently. Data in reports from a single subtype of periampullary adenocarcinoma may be confounded by inadvertent inclusion of tumours from other subtypes [6]. For example, inadequate exclusion of ampullary carcinomas from series of ductal pancreatic adenocarcinoma may lead to overestimation of long-term survival [10].

In addition to the commonly evaluated histopathologic factors, the histologic type of differentiation has been shown to have biologic and prognostic relevance for ampullary adenocarcinoma [6,7,11-14]. Kimura et al [13] were the first to demonstrate that adenocarcinomas originating in the ampulla of Vater may be classified as having either "intestinal" or "pancreatobiliary" type of differentiation, of which patients with the latter type consistently have been shown to have a worse prognosis [6,7,11-14]. This classification scheme is now widely accepted for ampullary adenocarcinoma and has also been suggested for extrahepatic bile duct carcinoma [15] and ductal pancreatic adenocarcinoma [16], but has not, to our knowledge, been applied previously as a basis for analysis of prognostic factors after periampullary adenocarcinoma resections. In the present study, we hypothesized that an evaluation of the histologic type of differentiation could independently predict the prognosis after periampullary resections and possibly give more precise information about patient prognosis than evaluation of tumour origin.

## Methods

### Patients

Permission for the study was obtained by the National Committees for Research Ethics in Norway. The patients included in the study comprised all patients (n = 213) with primary periampullary adenocarcinoma who underwent a pancreaticoduodenectomy with curative intent between 1980 and 2004 at Rikshospitalet University Hospital, a third-level referral hospital. In January 1998, the procedure for histopathologic reporting changed from a non-standardized procedure to a standardized procedure, in particular with respect to assessment of resection margins and tumour origin. Patients resected before and after the first of January 1998 were therefore assigned to a historical control group and a study group, respectively.

From 1998 to 2004 (study group), a total of 161 patients underwent pancreaticoduodenectomy, of which 114 patients had primary adenocarcinoma with macroscopically free margins (R0 or R1 resections). Excluded cases comprised patients with benign lesions (n = 22), neuroendocrine tumours (n = 9), invasive IPMN (n = 4), secondary carcinoma (n = 6), acinar cell carcinoma (n = 1), adenosquamous carcinoma (n = 1), and non-curative resection (i.e. macroscopic residual tumour, R2 resection; n = 4). Histopathologic features were analyzed in order to determine (1) whether the histologic type of differentiation is an independent prognostic factor in periampullary adenocarcinoma, and (2) to evaluate predictors of poor prognosis in the subgroup of patients that had a pancreatobiliary differentiated periampullary tumour.

Among the 114 patients in the study group, 82 were dead by the end of the study and the remaining 32 were followed for a median of 5.8 years (range 2.4–9.3). In the subgroup of patients with pancreatobiliary type adenocarcinoma, 58 (of 67) were dead by the end of the study, and the remaining 9 patients were followed median 6.1 years (range 3.5–8.7). The relatively many deaths and long follow-up time for the censored cases thus permitted a subgroup analysis of pancreatobiliary cases. Perioperative death (in-hospital death or death within 30 days of operation) was 3.5% (4/114) in the study group, among which three patients had ampullary tumour (3/41) and one had tumour originating in ductal pancreatic tissue.

The patients resected between 1980 and 1997 (historical control group, n = 99) provided a separate dataset in which to validate the main conclusions from the study group analysis. Tumour origin and differentiation type was compared in the two datasets obtained by standardized (study group) and non-standardized (historical control group) histopathologic evaluation, respectively. In the historical control group, 89 (of 99) patients were dead by the end of the study. The remaining 10 patients were followed median 12.4 years (range 9.4–20.8). In the subgroup of patients with pancreatobiliary type adenocarcinoma, 69 (of 73) were dead by the end of the study, and the remaining 4 patients were followed median 13.5 years (range 10.4–20.8). Perioperative death was 4.0% (4/99) in the historical control group. Cases with perioperative death were included in the survival analyses. No patients were lost to follow-up. Data from this series has been reported previously [12].

### Histopathologic assessment of specimens

In the study group, histopathologic factors were prospectively registered by routine examination according to a standardized, systematic protocol, and reevaluated retrospectively. The evaluated histopathologic factors were tumour origin, histopathologic type of differentiation, pT stage, maximum tumour diameter, resection margin involvement (with special attention to the retroperitoneal margin), perineural and vascular infiltration, regional lymph node involvement, and degree of differentiation.

Approximately 15 tissue samples were taken from each specimen including whole-mount blocks for most cases. A section parallel to the ampulla, distal bile duct, pancreatic duct, and parallel to the longitudinal duodenal axis was made in order to demonstrate the tumour's relation to each of these sites of potential tumour origin. The cancer origin was determined by tumour location relative to ductal anatomy and duodenal and pancreatic parenchyma, and by associated epithelial dysplasia or in situ neoplasia. Macroscopic pictures were also taken in selected cases.

**Figure 1:**
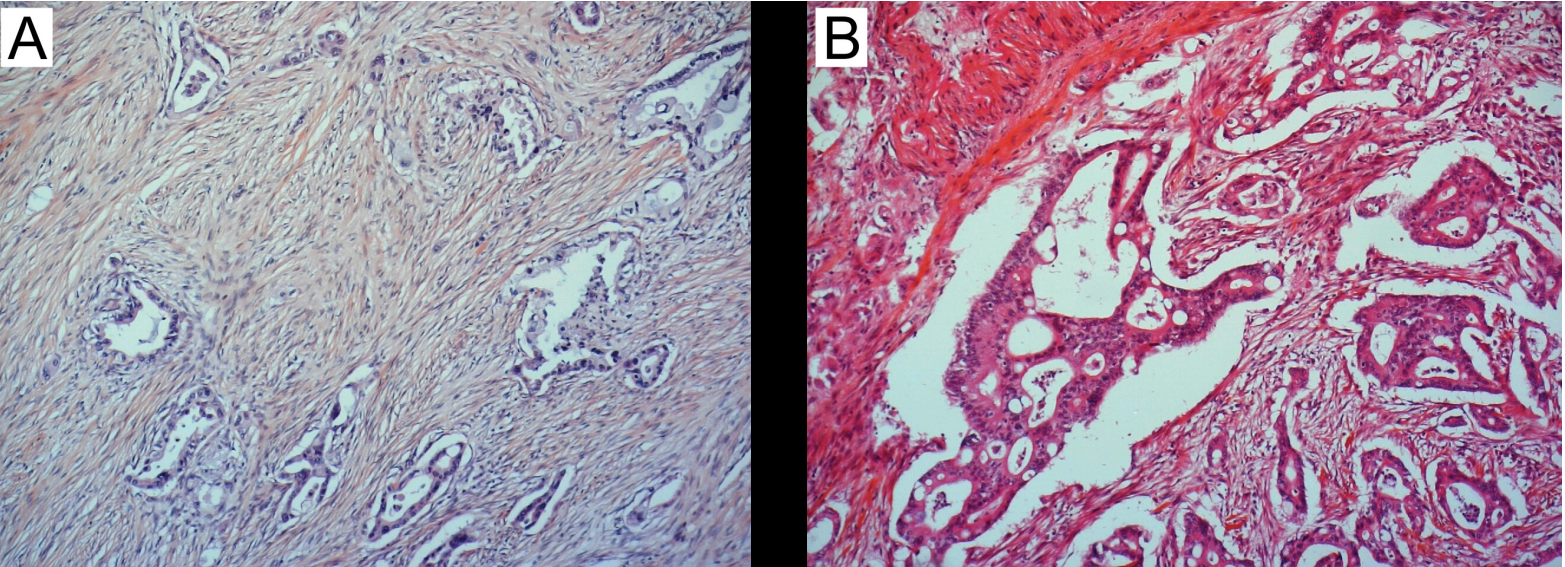
**The two dominant types of histologic differentiation in periampullary adenocarcinomas:** (A) The pancreatobiliary type typically has simple or branching glands and small solid nests of cells surrounded by a desmoplastic stroma, and cuboideal to low columnar epithelium arranged in a single layer without nuclear pseudostratification, the nuclei rounded but with marked variation in size and shape from one cell to the next. (B) The intestinal type typically resembles colon cancer, may consist of solid nests with cribriform areas, has tall and often pseudostratified columnar epithelium with oval nuclei located in the more basal aspects of the cytoplasm, and there may also often be presence of mucin.

The histologic type of differentiation was classified according to the criteria first suggested by Kimura et al [13], later revised by Albores-Saavedra et al [7] (figure 1). In brief, pancreatobiliary tumours typically have simple or branching glands and small solid nests of cells surrounded by a desmoplastic stroma, have cuboideal to low columnar epithelium arranged in a single layer without nuclear pseudostratification, and the nuclei are rounded but with marked variation in size and shape from one cell to the next. Intestinal tumours typically resemble colon cancer, may consist of solid nests with cribriform areas, have tall and often pseudostratified columnar epithelium with oval nuclei located in the more basal aspects of the cytoplasm, and there may also often be presence of mucin. Cases with mixed type differentiation were classified according to the dominant pattern, without performing cut-off optimalization prior to classification [17]. All tumours were assigned to one of these two histologic types of differentiation using this approach. In the historical control group (n = 99), cases with mixed type differentiation were identified (n = 13; 7 predominantly pancreatobiliary and 6 predominantly intestinal), and survival was compared between cases with mixed and single type of histologic differentiation in each group with respect to the predominant histologic pattern of differentiation. Patients with mixed type of differentiation had the same prognosis as patients with only the predominant type, eg. with only pancreatobiliary (p = 0.35) or only intestinal (p = 0.21) type of differentiation, respectively, thus indicating that classification based on the predominant pattern is applicable.

For both tumour origin and histologic type of differentiation, the prospective registrations were reevaluated independently by two experienced pathologists, a routine pathologist (ST) and a senior pathologist (OPFC). The routine pathologist reviewed only the microscopic slides and was blinded towards clinical and macroscopic data, while the senior pathologist also considered this information when determining the anatomic site of tumour origin. Upon disagreement, final determination of tumour origin and histologic type of differentiation was reached by a second reevaluation of the slides and by reevaluation of the histopathologic and operative reports.

In the historical control group (non-standardized protocol), all histopathologic reports and microscopic slides were reevaluated by a single pathologist (OPFC). The histopathologic factors registered in this group were tumour origin, histopathologic type of differentiation, maximum tumour diameter, resection margin involvement, perineural infiltration, regional lymph node involvement, and degree of differentiation. Only pancreatobiliary or intestinal adenocarcinomas were included in the analysis of histopathologic factors. R2 cases were not excluded from the analysis in the historical control group due to non-standardized reporting for this cohort.

### Statistical analysis

Survival data was obtained from the National Registry of Norway, updated May 30, 2007. The Kaplan-Meier method was used to calculate curves for overall survival and to estimate median survival. Survival curves were compared using the log-rank test. Associations between categorical variables were examined using Chi-square test. Mann-Whitney test was performed to compare maximum tumour diameter (measured as a continuous variable) between groups of independent samples. Interobserver agreement was estimated by Cohen's kappa and categorized as poor (kappa < 0.20), fair (0.21 < kappa < 0.40), moderate (0.41 < kappa < 0.60), substantial (0.61 < kappa < 0.80), or almost perfect (kappa > 0.80). Cox regression models were fitted in order to estimate univariate and multivariable survival, together with the hazard ratios and their 95% confidence intervals. For categorical variables, the group with the best prognosis in univariate analysis was set as reference. The multivariable survival model included all histopathologic factors, and the factors were further evaluated using stepwise variable selection. The model obtained from multivariable analysis in periampullary adenocarcinomas was tested in the ampullary subgroup in order to evaluate how well multivariable analysis with the same set of covariates could predict the prognosis in this group for which classification by histologic type of differentiation is already established. A separate multivariable Cox regression subgroup analysis was performed for patients with pancreatobiliary differentiated periampullary adenocarcinoma in order to determine the factors that were independently associated with survival in this subgroup. Finally, the main findings obtained from the study group analysis were validated by analysis of histopathologic factors in a historical control group.

Statistical analyses were performed in SPSS 15.0 for Windows software (SPSS Inc., Chicago, Illinois, USA). R version 2.3.1 (open source statistical software [18]) was used for testing goodness-of-fit based on martingale residual processes. For all tests, a two-sided p < 0.05 was considered statistically significant.

## Results

In the study group comprising 114 periampullary adenocarcinomas, there were 67 with pancreatobiliary and 47 with intestinal histologic type of differentiation. These consisted of 40 pancreatic, 41 ampullary, 17 common bile duct, and 16 duodenal adenocarcinomas. Interobserver agreement between the senior and routine pathologist was almost perfect (kappa 0.90; 95% CI 0.82–0.99) for determination of histologic type of differentiation, while it was only fair (kappa 0.37; 95% CI 0.25–0.49) in classification of tumour origin. However, while the routine pathologist who reevaluated the microscopic slides was blinded towards clinical and macroscopic data, the senior pathologist not only reevaluated the microscopic slides but also considered information from the operative and macroscopic reports. This type of information may be more important for accurate tumour origin classification than for histologic type classification. Thus, comparing final consensus with the original reports, interobserver agreement was substantial for both tumour origin classification (25 reclassified cases; kappa 0.68; 95% CI 0.57–0.79) and histologic type classification (13 reclassified cases; kappa 0.74; 95% CI 0.61–0.87).

### Histopathologic prognostic factors in periampullary adenocarcinoma

**Figure 2:**
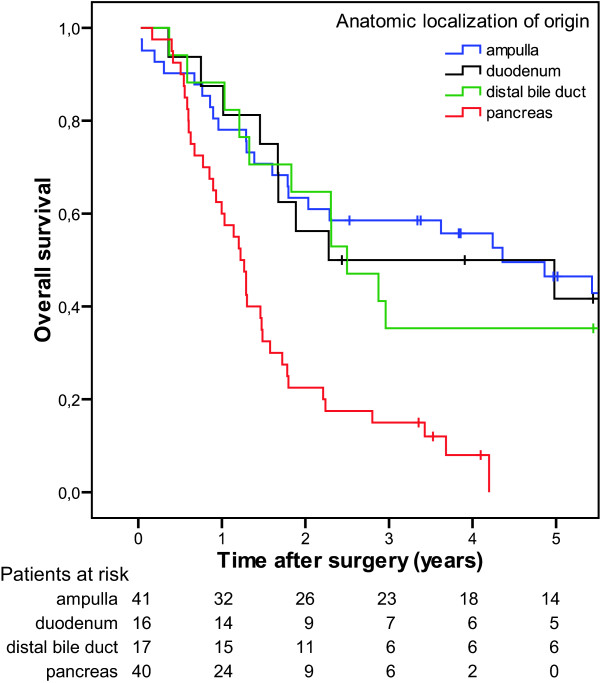
Overall survival after pancreaticoduodenectomy for periampullary adenocarcinoma (n = 114) originating in duodenum (n = 16), ampulla (n = 41), distal bile duct (n = 17), and pancreas (n = 40) (p < 0.001).

**Figure 3:**
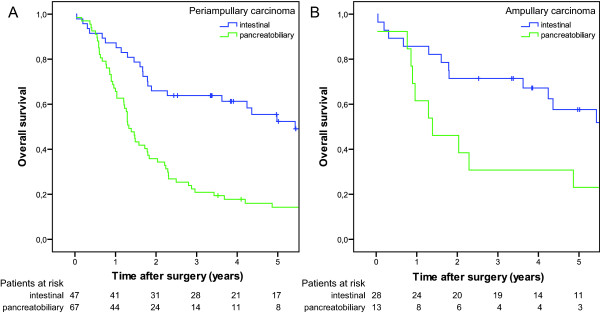
**Overall survival after pancreaticoduodenectomy:** (A) Periampullary adenocarcinoma (n = 114) with intestinal (n = 47) and pancreatobiliary (n = 67) type of histologic differentiation (p < 0.001). (B) Ampullary adenocarcinoma (n = 41) with intestinal (n = 28) and pancreatobiliary (n = 13) type of histologic differentiation (p = 0.02).

As expected patients with pancreatic tumours had the poorest prognosis among all periampullary adenocarcinomas in univariate analysis (p < 0.001, figure 2). Pancreatobiliary type of differentiation was an adverse predictor of survival both in the whole cohort of periampullary adenocarcinomas (p < 0.001, figure 3A) and in the ampullary subgroup (p < 0.022, figure 3B).

Table 1 describes associations between histologic type of differentiation and the other histopathologic factors. Compared to intestinal type adenocarcinomas, pancreatobiliary type adenocarcinomas significantly more often showed presence of histopathologic features associated with a poor prognosis, in particular resection margin involvement, perineural infiltration, areas with poor differentiation, advanced pT stage, and pancreatic tumour origin (p < 0.001 for each).

**Table 1:** Associations between histologic type of differentiation and other histopathologic factors in 114 periampullary adenocarcinomas

		Differentiation of adenocarcinoma		
		intestinal	pancreatobiliary	p-value^a^
Tumour origin	ampulla	28	13	< 0.001
	duodenum	16	0	
	distal bile duct	1	16	
	pancreas	2	38	
pT stage	pT1	10	2	< 0.001
	pT2	20	9	
	pT3	8	46	
	pT4	9	10	
Lymph node status	N0	23	26	0.282
	N1	24	41	
Resection margin status	R0	40	34	< 0.001
	R1	7	33	
Vessel involvement	free	35	36	0.025
	involved	12	31	
Perineural infiltration	no	35	17	< 0.001
	yes	12	50	
Areas with poorly differentiated tumour	no	39	32	< 0.001
	yes	8	35	
Tumour size (maximum tumour diameter)	small (≤ 2.5 cm)	31	35	0.108^b^
	large (> 2.5 cm)	16	32	

^a^Chi-square test, when not otherwise specified

^b^Mann-Whitney test (histologic type of differentiation vs tumour size measured as a continuous variable)

In multivariable analysis adjusting for tumour origin, pT stage, maximum tumour diameter, degree of differentiation, regional lymph node metastasis, resection margin involvement, vessel involvement, and perineural infiltration, the histologic type of differentiation was found to be an independent predictor of survival (p = 0.032; HR 2.8; 95% CI 1.1–7.1), while tumour origin was only borderline significant (p = 0.054). Stepwise backward variable selection resulted in a final model that included the histologic type of differentiation, which in fact was the strongest predictor of survival (table 2). The validity of the final model was tested in the ampullary subgroup, confirming that pancreatobiliary versus intestinal type of differentiation was an independent adverse predictor of survival also among these patients (p < 0.002; HR 4.0; 95% CI 1.6–9.6).

**Table 2:** Multivariable Cox regression analysis of histopathologic prognostic factors in periampullary adenocarcinomas (n = 114)

		HR	95% CI	p-value
Histologic type	pancreatobiliary (vs intestinal)	3.1	1.8–5.1	< 0.001
Lymph node involvement	N1 (vs N0)	2.5	1.5–4.4	< 0.001
Vessel involvement	involved (vs not involved)	1.9	1.2–3.1	0.012
Tumour size	diameter (measured in cm)	1.3	1.1–1.5	0.011

HR, hazard ratio. HR > 1 indicates increased probability of death compared to the reference group (for categorical variables) or compared to each increase of one cm in tumour size (continuous variable)

CI, confidence interval

### Prognostic factors in pancreatobiliary differentiated periampullary adenocarcinomas

**Figure 4:**
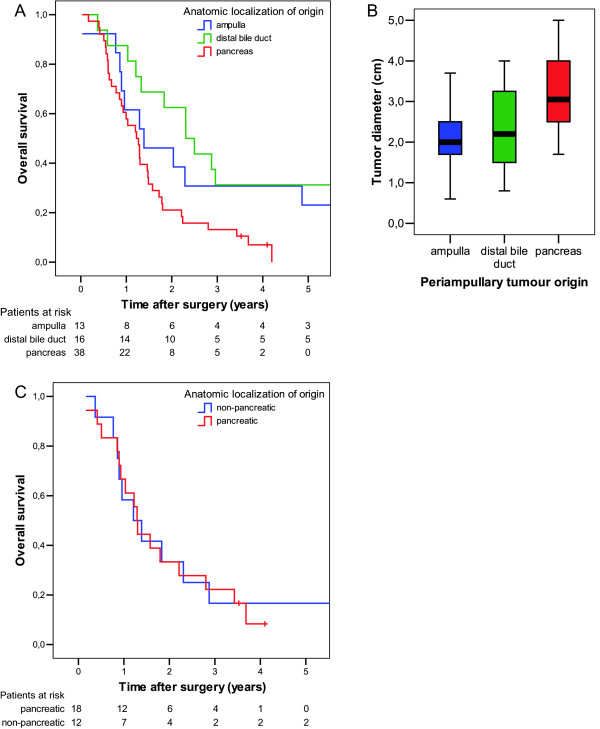
**Pancreaticoduodenectomy for pancreatobiliary type periampullary adenocarcinoma:** (A) Univariate survival for patients with pancreatobiliary type periampullary adenocarcinomas (n = 67) originating in ampulla (n = 13), distal bile duct (n = 16), and pancreas (n = 38) (p = 0.009). (B) Boxplot of maximum tumour diameter in the same 67 tumours with pancreatobiliary differentiation (p < 0.001, pancreatic versus non-pancreatic). (C) Survival for patients with non-pancreatic (n = 12, of which 6 ampullary and 6 biliary) and pancreatic (n = 18) tumours of comparable size (2 cm ≤ maximum diameter ≤ 3 cm) (p = 0.851).

A separate analysis of histopathologic factors among the patients who had pancreatobiliary adenocarcinoma (n = 67) was performed in order to identify prognostic factors in this subgroup, and in particular, to evaluate whether tumour origin could independently predict the prognosis in pancreatobiliary type adenocarcinoma. In univariate survival analysis, pancreatic tumour origin was significantly associated with a poorer prognosis compared to non-pancreatic tumour origin (table 3, figure 4A). Even when adjusting for pT stage, the difference in survival between patients who had pancreatic and non-pancreatic tumour origin was statistically significant (p = 0.003; HR = 2.9; 95% CI 1.4–6.0). However, adjusting for tumour diameter instead of pT stage demonstrated that there was in fact no survival difference between patients who had pancreatic and non-pancreatic tumours (p = 0.25). The pT staging for periampullary adenocarcinomas is based on the assumption that clinical outcome depends more on tumour extension beyond organ of origin than of tumour size. An ampullary pT3 tumour slightly invading the pancreas may thus be as small as 1 cm, while pancreatic pT3 tumours are normally much larger. In the present subgroup analysis of pancreatobiliary differentiated tumours, pancreatic pT3 tumours were significantly larger than non-pancreatic pT3 tumours (median diameter 3.5 versus 2.6 cm; p = 0.033).

**Table 3:** Survival analysis of histopathologic prognostic factors in pancreatobiliary resections (n = 67)

			p-value	
	HR	95% CI	Univariate	Multivariable
Pancreatic tumour origin (pancreatic vs non-pancreatic)	2.3	1.3–4.0	0.004	
pT stage (pT3 or pT4 vs pT1 or pT2)	2.0	0.9–4.2	0.073	
Resection margin involvement (R1 vs R0)	1.7	1.0–2.8	0.052	
Lymph node involvement (N1 vs N0)	3.0	1.6–5.4	< 0.001	0.007
Poor differentiation (yes vs no)	1.6	0.9–2.6	0.094	
Vessel involvement (yes vs no)	3.1	1.8–5.3	< 0.001	0.035
Perineural infiltration (yes vs no)	2.8	1.4–5.5	0.003	
Tumour size (continuous, measured in cm)	1.7	1.3–2.1	< 0.001	< 0.001

HR, hazard ratio. HR > 1 indicates increased probability of death

CI, confidence interval

Among all 67 pancreatobiliary differentiated adenocarcinomas, pancreatic tumours significantly more often than non-pancreatic tumours had regional lymph node metastasis (29/38 for pancreatic versus 12/29 for non-pancreatic tumours, p = 0.004) and were larger (median diameter 3.1 cm for pancreatic versus 2.0 cm for non-pancreatic tumours, p < 0.001) (figure 4B). The differences in survival seen among all cases (figure 4A) are thus related to the differences in tumour diameter (figure 4B) between non-pancreatic and pancreatic tumours. Selecting tumours of comparable size (range 2.0–3.0 cm, n = 30) demonstrated no difference in survival between pancreatic (n = 18) and non-pancreatic cases (n = 12) (p = 0.851, figure 4C). These groups were comparable with respect to tumour diameter (median 2.5 and mean 2.4 cm for both groups), and the equal survival was not due to less frequent lymph node metastasis among pancreatic cases (positive lymph nodes in 15/18 pancreatic compared to 6/12 non-pancreatic cases).

Starting with all the histopathologic factors in the base model for multivariable analysis, backward variable selection thus resulted in a final model that did not include tumour origin (table 3). Only lymph node status, vessel involvement and tumour diameter independently predicted the prognosis after resection of pancreatobiliary type periampullary adenocarcinoma. The final model obtained from stepwise backward analysis was confirmed by repeating variable selection with forward stepwise analysis. Although perineural infiltration also seemed to be an important prognostic factor in univariate analysis, this factor did not independently predict survival, due to a strong association with lymph node metastasis (p = 0.002), vessel involvement (p < 0.001), and tumour diameter (p = 0.024).

### Validation of main conclusions in an independent dataset

We finally validated our main findings by performing a separate analysis of histopathologic prognostic factors in the historical control group consisting of patients operated in our institution before standardization of histopathologic assessment. Among these patients, 73 and 26 were upon reevaluation of the histologic slides found to have pancreatobiliary and intestinal differentiation, respectively. Tumour origin for all 99 cases was classified as ampullary (n = 23), duodenal (n = 14), distal bile duct (n = 10), and pancreatic (n = 52).

In univariate survival analysis, pancreatobiliary type of differentiation (p < 0.001) and pancreatic tumour origin (pancreatic versus ampullary, p = 0.03) both predicted a poor prognosis. Adjusting for maximum tumour diameter, lymph node and resection margin involvement, degree of differentiation, and whether there was presence of perineural infiltration, the histologic type of differentiation remained highly significant (p < 0.001; HR 2.7; 95% CI 1.5–4.9). In contrast, although approaching significance, tumour origin did not significantly predict the prognosis after adjustment for these other factors (pancreatic versus ampullary, p = 0.10; HR 1.6; 95% CI: 0.9–2.9).

In the subgroup analysis including only pancreatobiliary differentiated tumours (n = 73), stepwise variable selection resulted in a final multivariable model in which lymph node status (N1 versus N0, p < 0.001; HR 3.4; 95% CI: 1.9–6.0) was confirmed to independently predict the prognosis, adjusting for grade of differentiation (high/moderate versus low, p = 0.002; HR 2.6; 95% CI 1.4–4.8) and resection margin status (R1/2 versus R0, p = 0.011; HR 1.9; 95% CI 1.2–3.2). Although significant in univariate analysis (p = 0.021), tumour diameter was not confirmed to independently predict survival in this cohort, possibly due to the small size of these tumours (mean diameter 2.1 cm; 95% CI 1.9–2.4) and a significant association between tumour diameter and lymph node status (p = 0.04). Finally, after stepwise variable selection, tumour origin and perineural infiltration did not remain in the final multivariable model evaluating histopathologic factors of pancreatobiliary differentiated periampullary adenocarcinomas, in accordance with the results from the study group analysis.

## Discussion

In the present study, we evaluated the prognostic importance of the two main histologic types of differentiation, pancreatobiliary and intestinal types, in presumed curative resections for pancreatic head adenocarcinomas. We found that the pancreatobiliary histologic type of differentiation was independently associated with a poor prognosis, while tumour origin did not significantly predict survival when adjusting for other histopathologic prognostic factors. In pancreatobiliary type adenocarcinomas, survival depended on factors related to the disease stage (tumour size and regional lymph node involvement), but not on the anatomic structure of origin. The main conclusions from this study were confirmed by analysis in an independent dataset.

For multiple reasons, we suggest that determination of the histologic type of differentiation is a useful adjunct to classification of the anatomical site of origin in periampullary tumours. Failure to reach a precise diagnosis of tumour origin may lead to false assumptions regarding long-term survival [2,7,10]. Periampullary anatomy may be distorted by carcinoma or affected by inflammation and fibrosis [9]. The normal ampulla, defined as the junction between the distal bile duct and the main pancreatic duct, has a variable length and is absent in a large proportion of the normal population [9]. Resected ductal pancreatic adenocarcinomas in the pancreatic head typically have a mean diameter of ~3 cm[1], and may involve the entire ampullary region [5]. Determination of tumour origin should therefore be standardized and include a section parallel to (and including) the ductal structures in order to evaluate the tumour's relation to the ductal anatomy. However, even with standardized evaluation, interobserver variability may be considerable unless clinical and macroscopic data is also emphasized.

The present study is the first report on the distribution of pancreatobiliary and intestinal type differentiation in a cohort of resected adenocarcinomas of all periampullary locations. With respect to ampullary tumours, it has been known for more than a decade that these may have either "intestinal" or "pancreatobiliary" histologic type of differentiation [13], and that the intestinal type has a significantly better prognosis [7,11-14]. Intestinal type biliary tract [15] and pancreatic [16] carcinomas have also been reported, but there is sparse data comparing pancreatobiliary type adenocarcinomas of these three different origins [19-21]. Although many studies have compared ductal adenocarcinomas of different periampullary origin [19-22], most studies do not state specifically whether intestinal type ampullary adenocarcinomas were excluded in such comparisons. It should be noted that ductal adenocarcinoma is not synonymous with pancreatobiliary type of histology, since it has recently been shown that ductal pancreatic carcinomas may have features of intestinal differentiation [16]. Pancreatobiliary ampullary carcinomas that involve the pancreas may be indistinguishable from pancreatobiliary pancreatic carcinomas that extend into the ampulla [2,8], and low ratios of ampullary versus periampullary adenocarcinomas in many studies might reflect a tendency towards misclassification of advanced pancreatobiliary type ampullary carcinomas as ductal pancreatic carcinomas.

An explanation for the often reported more favourable prognosis in non-pancreatic versus pancreatic carcinoma could therefore be that comparison has not been strictly limited to pancreatobiliary type adenocarcinomas. The reason why there are prognostic differences between equally advanced non-pancreatic and pancreatic adenocarcinomas may simply be that the latter more often has a pancreatobiliary type of histologic differentiation [7]. Thus, the question whether survival differences between pancreatic and non-pancreatic pancreatobiliary type adenocarcinomas should be attributed to disease stage or biology, or both, has not been definitely answered [2]. The present study suggests that survival differences between periampullary adenocarcinomas of comparable size are more dependent on the histologic type of differentiation than on the anatomic origin.

In the UICC/AJCC classification of pancreatic head malignancies, adenocarcinomas originating from the peri-Vaterian duodenum, the ampulla of Vater, the distal bile duct, and the ductal pancreatic tissue are considered separate entities [23,24], and the TNM staging criteria are different for each origin. However, since adenocarcinomas arising from the peri-Vaterian duodenum and the ampulla of Vater may be indistinguishable [7], and since it may also be difficult to discriminate between pancreatobiliary differentiated ampullary and distal bile duct adenocarcinomas [15], pT stage might be classified according to inappropriate anatomic location. Furthermore, pT1 in ductal pancreatic carcinoma includes tumours with maximum diameter up to 2 cm, while pT1 in ampullary carcinomas includes only small tumours not invading either the duodenum (pT2) or the pancreas (pT3). A relatively large pancreatic tumour could therefore still be at pT1 stage compared to a small ampullary tumour extending into the duodenum, thus classified as pT2 (or even extending into the pancreatic tissue, thus classified as pT3). Importantly, pT stage was the factor with the lowest p-value both in univariate and multivariable survival analysis of all periampullary adenocarcinomas as well as in the subgroup of pancreatobiliary differentiated adenocarcinomas. The present study therefore demonstrates that individual factors related to tumour stage are more reliable than pT (or TNM) stage group in multivariable analysis of prognostic factors in pancreatic head carcinomas.

## Conclusion

Pancreatobiliary versus intestinal type of differentiation independently predicts poor prognosis after pancreaticoduodenectomy for periampullary adenocarcinoma. In pancreatobiliary type periampullary adenocarcinoma, lymph node involvement, vessel involvement, and increasing tumour diameter were adverse predictors of survival in the present study.

## Competing interests

The authors declare that they have no competing interests.

## Authors' contributions

AW participated in design of the study, registration and ethical approval, patient inclusion, review of clinical data, and histopathologic analysis. He also designed the database, performed the statistical analysis, and drafted the manuscript. ST participated in patient inclusion and had a major responsibility for histopathologic analysis. INF contributed with establishment of the protocol for systematic histopathologic assessment, participated in design of the study, patient inclusion, and histopathologic analysis. MC contributed substantially with choice of statistical methods and participated in statistical analysis. TJE participated in establishing systematic pathologic review of pancreaticoduodenectomy specimens, in design of the study, and in histopathologic analysis. ØM contributed substantially in the discussion of operative methods and performed many of the pancreaticoduodenectomies. OPFC participated in design of the study, had a major responsibility for histopathologic analysis, and contributed substantially with critical review of the manuscript. IPG participated in design, registration and ethical approval of the research project, and in patient inclusion and registration of clinical data. He also performed many of the pancreaticoduodenectomies, contributed substantially in the discussion of statistical methods, and drafted the manuscript. All authors critically reviewed the manuscript and approved the final manuscript.

## Pre-publication history

The pre-publication history for this paper can be accessed here:


